# Supplementary material for: RNA helicase, DDX27 regulates skeletal muscle growth and regeneration by modulation of translational processes
Source: PLoS Genet. 2018 Mar 8;14(3):e1007226. doi: 10.1371/journal.pgen.1007226 (PMC5843160; doi:10.1371/journal.pgen.1007226)
Supplement: S1 Data — (DOCX) [file pgen.1007226.s001.docx]

**METHODS**

**ENU screen**

AB males were treated with 3 mM ENU once a week for 3 weeks. The males were then crossed repeatedly to clean out any post meiotic germ cells that were mutagenized. Mutagenized males were then crossed to wild-type AB females and the progeny (F1) were raised. F2 embryos were obtained by setting up at least six pairs of F1 crosses and screened for skeletal muscle defects. Zebrafish larvae were screened at 3–4 dpf using a birefringence assay. Crosses in which 25+5% of larvae that showed patchy or reduced birefringence of their axial skeletal muscles in polarized light were identified as potential skeletal muscle mutants and these families were selected for further study [59].

**Genetic mapping**

AB strain *ddx27*-mutated heterozygous zebrafish were outcrossed to wild-type wik to generate polymorphic mapping strains. Low-resolution mapping was done with 40 diploid mutant and 40 diploid wild-type embryos obtained from in-crossing mapping F2 fish. Microsatellite CA markers throughout the genome were used to scan for linkage.

**RNA extraction and RT–PCR**

RNA was isolated from pools of mutant or control embryos at different developmental stages or C2C12 cells by using RNeasy fibrous tissue mini kit (Qiagen). cDNA was prepared using superscript III first-strand synthesis kit (Invitrogen). Equal concentrations of control and mutant RNA were used for cDNA synthesis. qRT-PCR was performed using Sybr green dye assay (Kapa Biosystems). The sequence of the primers is described in supplemental table 1. The fold change quantification was performed using the 2^−ΔΔ^*^Ct^* method.

**Immunofluorescence**

Immunofluorescence was performed on frozen sections as described previously [26] with Pax7 (1:10, DHSB) and Ddx27 (1:20, Santa Cruz, sc-81074).

**Western blotting**

One hundred embryos of control or mutant fish at 5 dpf were extracted with 50 mM Tris–HCl buffer containing 150 mM NaCl and 2% Triton X-100. Following centrifugation at 14 000g at 48°C for 20 min, the concentration of protein in supernatants was determined by the Bradford method, using a protein assay kit (Bio-rad). Proteins were separated by electrophoresis on 4–12% gradient Tris–glycine gels (Invitrogen) and transferred onto polyvinylidene difluoride membranes (Invitrogen). Membranes were blocked in 5% nonfat dry milk in 1X PBS for 1h at RT and primary antibody at 4°C, overnight. After washing, the membranes were incubated with horseradish peroxidase secondary antibody (anti-mouse IgG, 1:5000,Thermo Fisher Scientific). Proteins were detected using a western blotting detection kit (Millipore).

**Creating knockout *Ddx27* C2C12 cell lines**

C2C12 cells were transfected with a pool of CRISPR plasmids targeting three different sites in mouse *Ddx27* gene. Each Plasmids contained Cas9, GFP and guide RNA targeting specific sites (Santa Cruz Biotechnology). GFP expressing transfected cells were purified by FACS sorting and single cells were plated in to 96 well plates. Following clonal expansion, individual clones were screened by genomic PCR and Sanger sequencing to identify homozygous mutations in *Ddx27*.

**Electron microscopy**

Zebrafish embryos were fixed in formaldehyde–glutaraldehyde– picric acid in cacodylate buffer overnight at 48C followed by osmication and uranyl acetate staining. Subsequently, embryos were dehydrated in a series of ethanol washes and finally embedded in Taab epon (Marivac Ltd., Nova Scotia, Canada). Ninety-five nanometer sections were cut with a Leica ultracut microtome, picked up on 100 m formvar-coated Cu grids and stained with 0.2% lead citrate. Sections were viewed and imaged under the Philips Tecnai BioTwin Spirit Electron Microscope (Electron Microscopy Core, Harvard Medical School).

**qRT PCR Zebrafish Primers**

The following qRT-PCR primers were used to quantify mRNA expression in zebrafish.

| **Primer Name** | **Sequence** |
| --- | --- |
| *ddx27* | Forward: 5’-CAG TAT TGA CGG CAT TCT GGG AG-3’  Reverse: 5’-TCT CTG CTT TCT CTT GAG CGG C-3’ |
| *mylz2* | Forward: 5’-TTG ACC ACT CAG TGC GAC AGG TTC-3’  Reverse: 5’-AAC ATT GCC AGC CAC ATC TGG G-3’ |
| *β-actin* | Forward: 5’-CGA GCA GGA GAT GGG AAC C-3’  Forward: 5’-CAA CGG AAA CGC TCA TTG C-3’ |
| *pax3a* | Forward: 5’-ATC ACA GGT GAT GGG GCT GTT-3’  Reverse: 5’-GTG CTG GTA TTC ACC TTG CTC-3’ |
| *pax3b* | Forward: 5’-TGT GAC CGG AAC AAC GTA CC-3’  Reverse: 5’-GAT GGA CTC GAT CTG TCC GC-3’ |
| *pax7a* | Forward: 5’-CGG GGA TAA AGG TAA TCG CA-3’  Reverse: 5’-TCA TGC ACT GTA AGT AGC GCA-3’ |
| *pax7b* | Forward: 5’-CCA CAA GAC GTC AGT GGC A-3’  Reverse: 5’-TCT CTG CTT TCT CTT GAG CGG C-3’ |
| *myf5* | Forward: 5’-CCA GAC AGT CCA AAC AAC AGA CC-3’  Reverse: 5’-TGA GCA AGC AGT GTG AGT AAG CG-3’ |
| *myod1* | Forward: 5’-TCG GGA TCT GAA GGA CTT TG-3’  Reverse: 5’-AAG CAA GGG TCG TCG TAG AA-3’ |
| *myoG* | Forward: 5’-GTG GAC AGC ATA ACG GGA ACA G-3’  Reverse: 5’-TCT GAA GGT AAC GGT GAG TCG G-3’ |
| *des* | Forward: 5’-CGA GAT TGA CTC TCT CAA GGG CAC -3’  Reverse: 5’-GGG CGA TAG TGT CCT GAT AAC CAC-3’ |
