# Supplementary figures and images for: RNA helicase, DDX27 regulates skeletal muscle growth and regeneration by modulation of translational processes
Source: PLoS Genet. 2018 Mar 8;14(3):e1007226. doi: 10.1371/journal.pgen.1007226 (PMC5843160; doi:10.1371/journal.pgen.1007226)

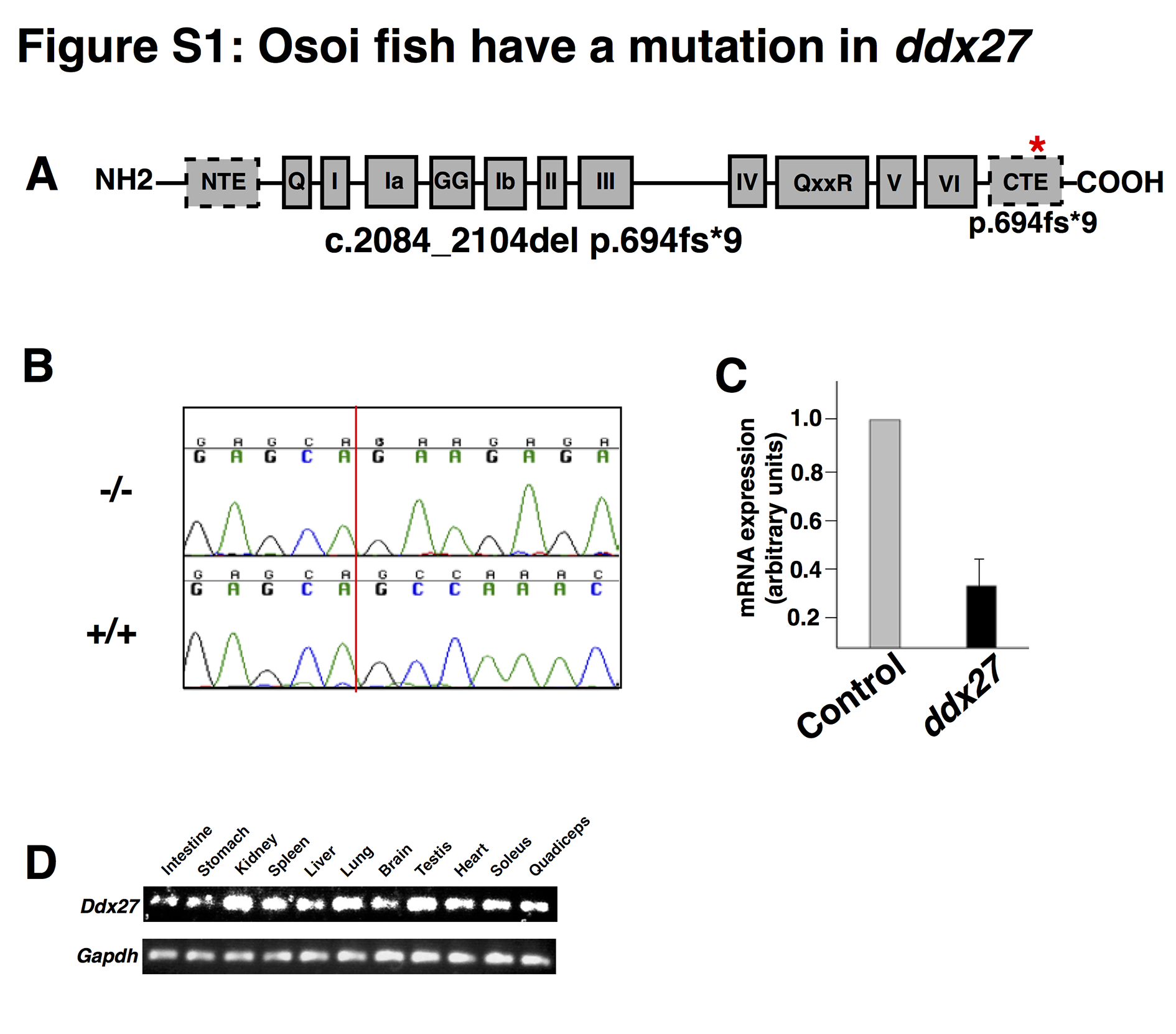

Supplement: S1 Fig — (A) The schematic diagram of DDX protein domains. DEAD-box helicases share a conserved core consists of two globular RecA like domains (Domain 1 and 2) that are involved in RNA binding and unwinding: Motifs I, II (DEAD) and VI are implicated in ATP binding and hydrolysis, Motif III couples ATP hydrolysis to RNA unwinding and Motifs IV, V and VI contribute to RNA binding. The variable N- and C-terminal extensions (NTE and CTE) provide specificity to each of the helicases by protein-protein interactions. p.694fs*9 mutation in Osoi fish is localized to CTE. (B) Sanger sequencing of candidate genes identified a 20 base pair deletion in open reading frame of ddx27 gene causing a frame shift mutation predicted to produce a truncated protein. (C) q RT-PCR analysis showed significantly reduced levels of ddx27 transcripts in mutant fish. (D) Tissue-wide expression analysis of Ddx27 in murine tissues by RT-PCR. GAPDH was used as a control. (TIF) [file pgen.1007226.s002.tif]

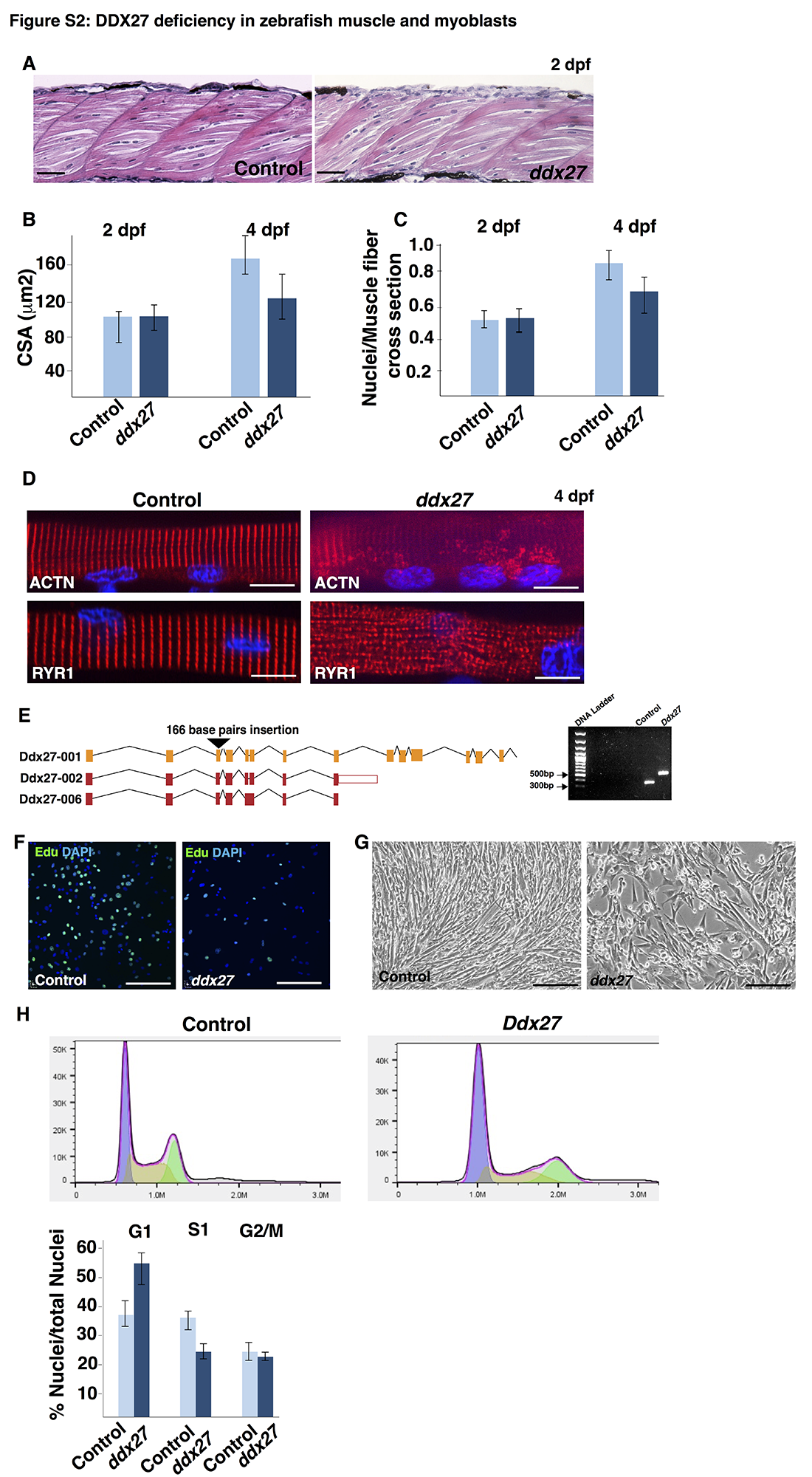

Supplement: S2 Fig — (A) Hematoxylin and eosin staining of embryonic skeletal muscle (2dpf) in control and ddx27 zebrafish. (B) Skeletal muscle growth during embryogenesis (2 dpf) and larval stages (4 dpf) was assessed by measuring cross-section area of myofibers in control and mutant fish. (C) Myonuclear content in control and ddx27 mutant fish was evaluated by quantifying the number of nuclei/myofiber in muscle cross-sections. 5 different areas in myotome were analyzed (n = 4). (D) Control and ddx27 zebrafish myofibers (4 dpf) were cultured and immunofluorescence analysis was performed. Expression of sarcomeric α-actinin labeling Z-line was reduced in mutant myofibers. Expression of sarcoplasmic reticulum marker, Ryr1 showed a disorganized pattern in comparison to control myofibers (scale bar: 100μm). (E) Three different guide RNAs (sgRNA) were designed targeting mouse Ddx27 gene. sgRNA targeted to exon3 of all three Ddx27 transcripts resulted in a 166 base pair homozygous insertion and generation of several stop codons. PCR analysis of genomic DNA revealed an insertion in exon3 of Ddx27 gene. (F) Control and Ddx27 mutant C2C12 were plated at equal concentration and grown in the proliferation media. Cells were pulsed treated with Edu (FITC signal) and counterstained with DAPI. (G) Control and Ddx27 mutant C2C12 cells were plated at equal cell density and grown in the differentiation media for 7 days. Control cells differentiated in to well differentiated myotubes/myofibers whereas Ddx27 cells exhibited a severe differentiation defects (scale bar: 50μm). (H) Cell cycle analysis in control and Ddx27 myoblasts. (TIF) [file pgen.1007226.s003.tif]

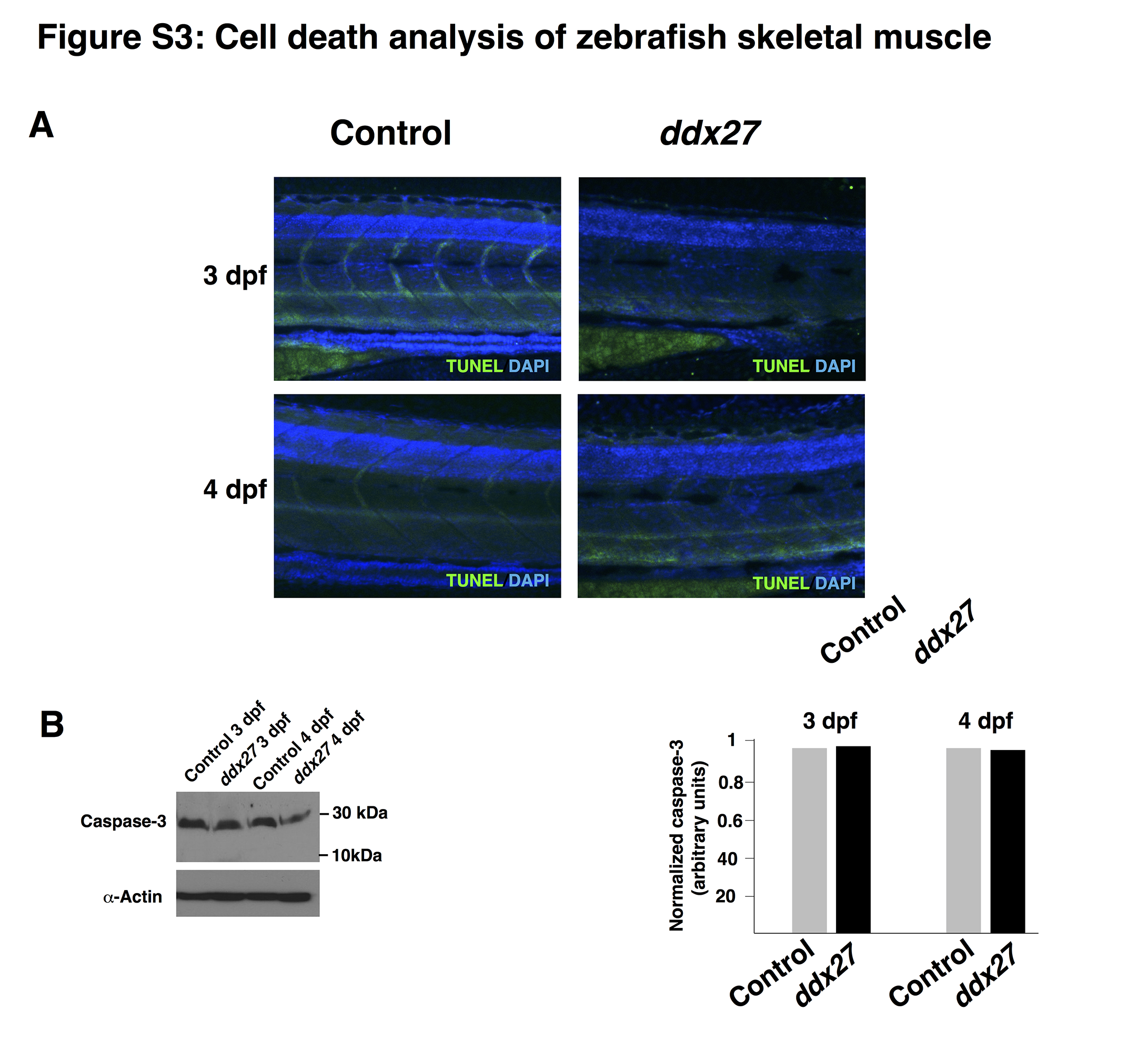

Supplement: S3 Fig — (A) Whole mount TUNEL labeling was performed in WT and ddx27 mutant zebrafish (3 and 4 dpf, n = 20) and myotome was analyzed. (B) protein extracts were prepared from control and ddx27 mutant fish (3 and 4 dpf) and western blot analysis was performed with caspapse 3 antibody. Quantification of western blot revealed no significant differences in cell death in control and mutant zebrafish. (TIF) [file pgen.1007226.s004.tif]

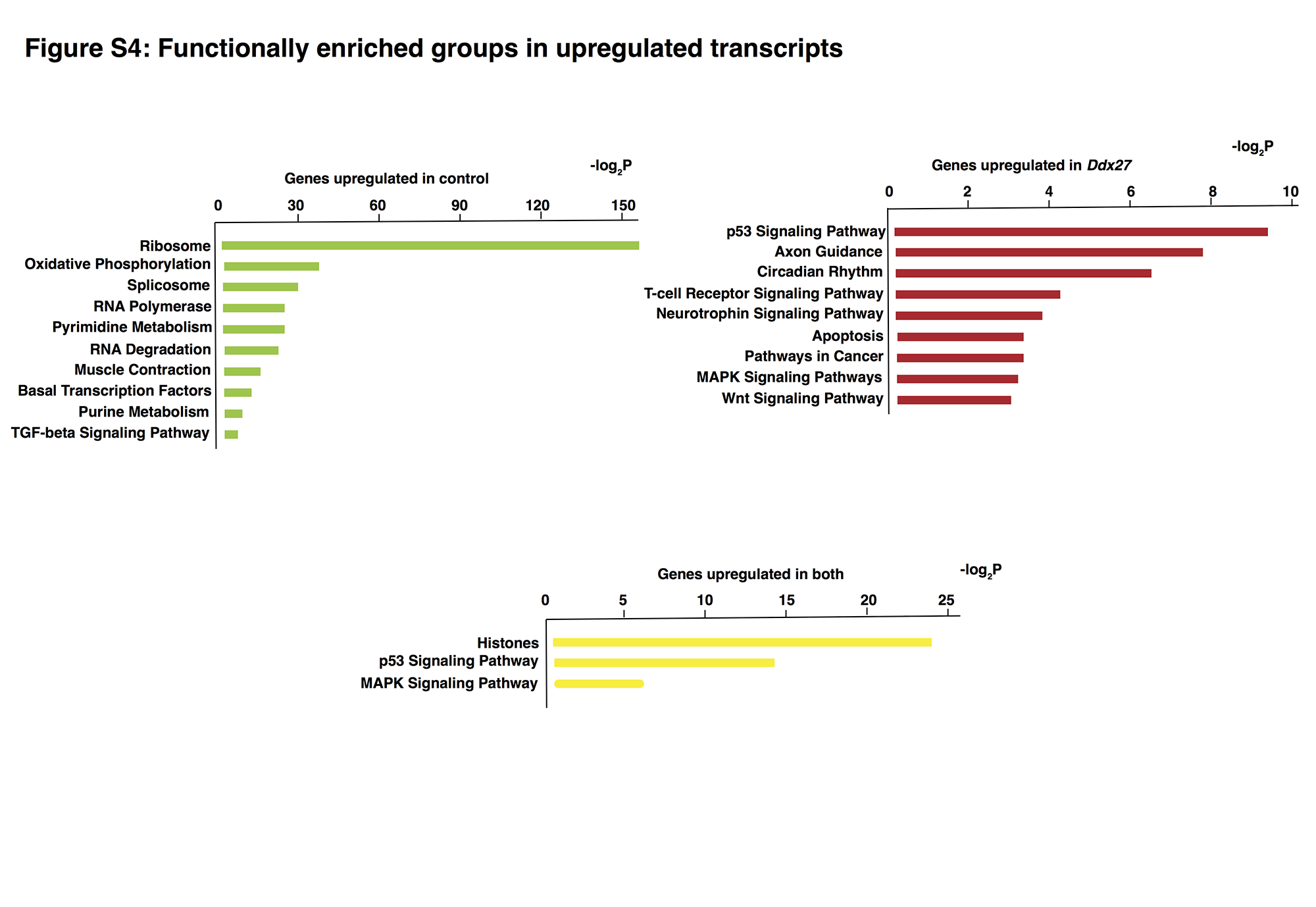

Supplement: S4 Fig — Kyoto encyclopedia of genes and genomics (KEGG) pathway analysis within the target genes of significantly altered mRNA was performed using the database for annotation, visualization and integrated discovery (DAVID) bioinformatics tools. The enriched KEGG pathways were identified and listed according to their enrichment p-values. (TIF) [file pgen.1007226.s005.tif]

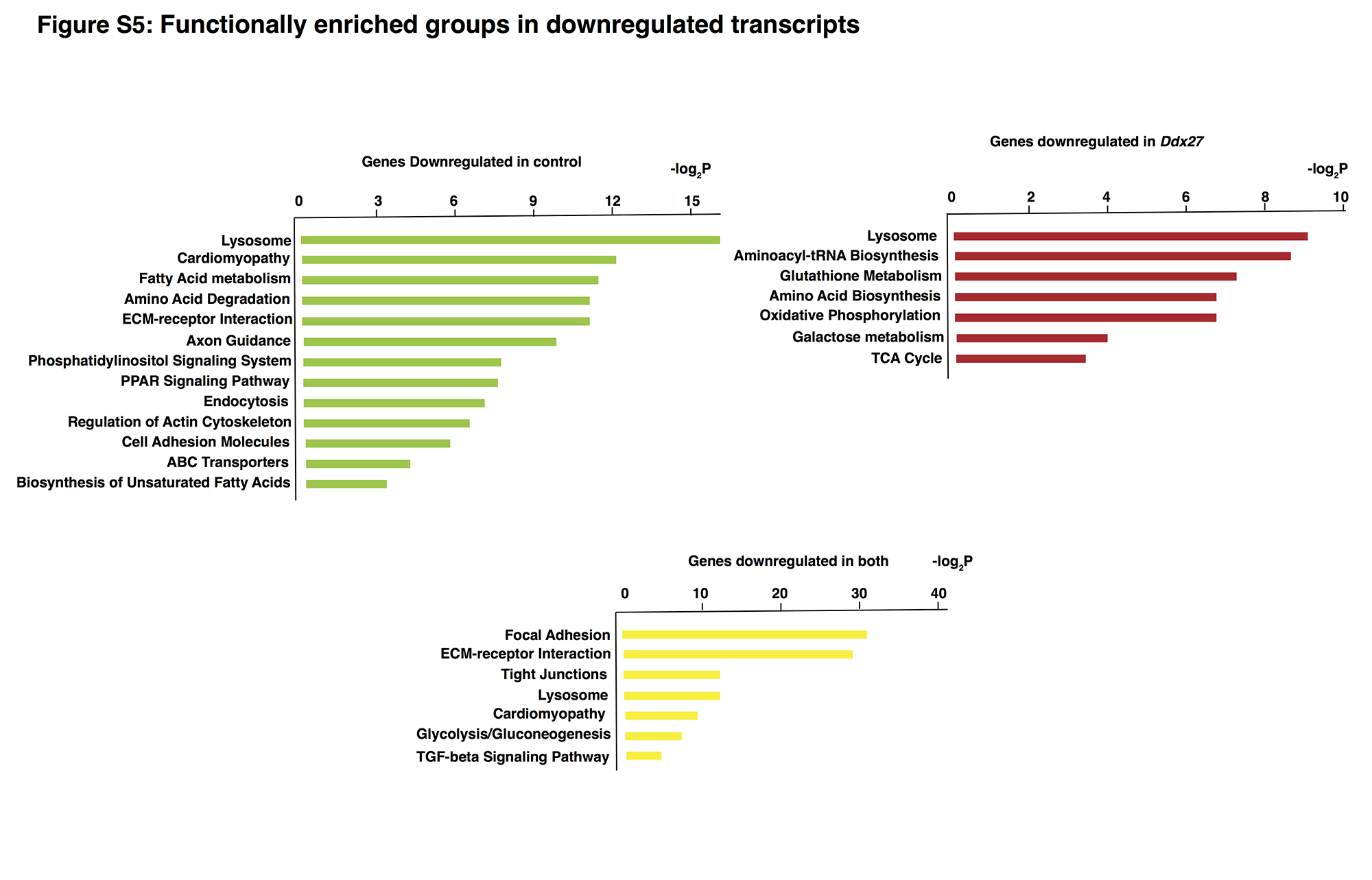

Supplement: S5 Fig — Kyoto encyclopedia of genes and genomics (KEGG) pathway analysis within the target genes of significantly altered mRNA was performed using the database for annotation, visualization and integrated discovery (DAVID) bioinformatics tools. The enriched KEGG pathways were identified and listed according to their enrichment p-values. (TIF) [file pgen.1007226.s006.tif]
